# Investigating the instability of variable selection in Lasso-penalized Cox proportional hazards models: a simulation study on low-dimensional real-world medical data

**DOI:** 10.1186/s12874-026-03019-4

**Published:** 2026-10-24

**Authors:** Bartosz Dobies, Dörte Huscher

**Affiliations:** 1 Doctoral School of Medical and Health Sciences, Jagiellonian University, ul. św. Łazarza 16, Kraków, 31-530 Poland; 2 Charité – University Medical Center Berlin, corporate member of Freie Universität Berlin and Humboldt-Universität zu Berlin, Institute of Biometry and Clinical Epidemiology, Charitéplatz 1, Berlin, 10117 Germany

**Keywords:** Lasso, Cox model, Survival analysis, Cross-validation, Variable selection, Simulation study, Hyperparameter tuning

## Abstract

**Background:**

This study examined the stability of Lasso-penalized Cox proportional hazards models in low-dimensional clinical data. While Lasso is a popular method for simultaneous variable selection and regularization, its performance is known to vary across cross-validation (CV) runs due to random fold assignments. We aim to quantify the effects of the number of folds, stratification procedures, and elastic net mixing parameters on variable selection consistency.

**Methods:**

We analyzed real-world data from the Berlin Initiative Study in older adults, focusing on a subcohort with measurements of a novel biomarker (*n* = 930) and assessing two time-dependent outcomes: all-cause mortality (*n* = 882) and a cardiovascular composite endpoint (CE, *n* = 638). We applied Lasso to identify prognostic variables among 20 medically preselected predictors. In simulation studies, we systematically varied the number of CV folds, implemented stratification by nonproportional covariates, and adjusted the elastic net’s mixing parameter. We evaluated model stability by examining variability in the number of selected predictors and predictive performance via the C-index.

**Results:**

In our datasets, the number of CV folds substantially influenced model size and selection stability across random seeds, with higher fold numbers generally yielding more consistent results (optimal: 10-fold for mortality, 15 for CE). Stratifying by covariates, which violated the proportional hazards assumption, enhanced stability in some cases. The elastic net did not consistently outperform Lasso in terms of stability or predictive accuracy; mixing parameters below 0.6 led to greater variability.

**Conclusions:**

The stability of Lasso-penalized Cox models is strongly affected by CV design. Higher fold numbers and appropriate stratification can improve stability, but at a computational cost. Elastic net offers no clear benefit in this setting. These findings highlight how routine modelling choices, including default cross-validation settings, may influence variable selection stability in low-dimensional clinical survival analyses.

**Supplementary Information:**

The online version contains supplementary material available at https://doi.org/10.1186/s12874-026-03019-4.

## Background

The growing prevalence of large datasets has made regularization and variable selection indispensable tools in statistics. The Least Absolute Shrinkage and Selection Operator (Lasso), introduced by Tibshirani [1], stands out for its ability to address both objectives simultaneously. The significance of this algorithm is underscored by the ongoing development of new extensions such as elastic net [2], adaptive Lasso [3], Bayesian Lasso [4], sparse-group Lasso [5], or prior Lasso [6].

Lasso has also gained widespread popularity in medical data analysis, particularly in studying biomarkers and gene expressions [7–9]. It can be implemented within the Cox proportional hazards model [10], a widely used survival analysis model in medical research for assessing the association between an individual’s survival time and explanatory variables. Lasso-penalized Cox regression enables the identification of the most important prognostic factors while analyzing survival data. The magnitude of shrinkage applied to the coefficients is controlled by the tuning parameter λ, which determines the strength of the penalty applied to regression coefficients. The optimal value for this parameter is found through resampling methods, with cross-validation (CV) standing as the predominant approach [11–13].

Despite its sparsity and interpretability, Lasso exhibits reduced algorithmic stability, making it susceptible to small perturbations in the input data [14]. The CV procedure, which relies on random allocation of observations to folds, may produce different tuning parameters across random seeds and thereby influence model results. Sensitivity to hyperparameter tuning has been reported in previous studies [15–18], although most investigations focus on high-dimensional settings (*p* > *n*) [19]. Penalized regression, however, is increasingly applied in clinical survival studies with moderate numbers of predictors, particularly when evaluating the independent contribution of potentially prognostic biomarkers [20–22].

This paper explores the stability of Lasso-penalized Cox proportional hazards models in real-world, low-dimensional medical data comprising 930 individuals. Through a comparison of cross-validated Lasso (Lasso-CV) and bootstrapped backward elimination results for two similar outcomes, we uncovered inconsistencies generated by Lasso-CV. To address this issue, we employ simulations to investigate the underlying causes of this instability, examining how the number of CV folds, stratification procedure, and incorporation of elastic net’s mixing parameters influence the variability in model size, but also the λ parameter and predictive performance. Additionally, we explore the possibility of mitigating this variability and enhancing the stability of Lasso-based variable selection.

## Methods

### Dataset

The Berlin Initiative Study, a prospective, population-based cohort study of individuals aged 70 and above, conducted a sub-analysis of a new biomarker involving 930 participants. Over a median follow-up period of 48.3 months, 70 strokes, 38 myocardial infarctions, and 234 deaths occurred. The analysis aimed to assess the prognostic value of 20 knowledge-based predictors, including the novel biomarker, for the outcomes of mortality and the composite endpoint (CE) of death and cardiovascular events (excluding cases with prior events) using Cox regression models [23].

The mortality dataset contains 882 observations with 218 events of interest and a median follow-up time of 59.6 months. The composite endpoint set includes 638 observations with 178 events and a median follow-up of 52.9 months. The average participants’ age in the mortality dataset was 82.2 years, and in the CE dataset 81.6 years. The mortality dataset includes 413 men (46.8%) and 469 women (53.2%), while the CE dataset exhibits a slight gender imbalance, with 264 men (41.4%) and 469 women (58.6%).

Two variable selection methods were employed to achieve model sparsity for both data settings and identify only the essential predictors: the Cox model with backward elimination in a bootstrap approach (BBE) with 500 bootstrap samples, and the Cox model with Lasso-CV across 500 random seeds.

### Cross-validation

The choice of optimal tuning parameter λ can be determined through the CV process, where the data is divided into *k* approximately equal-sized subsets called folds. One of the folds is used as a holdout set, and the remaining *k-*1 folds are used to fit candidate models. Through an iterative process, the candidate models are fitted to the remaining *k-*1 folds until each of the *k* folds has been left out. The optimal value of λ is chosen to be the one with the smallest CV error based on the sequence of λs estimated within the candidate models. The type of loss used for the cross-validated penalized Cox model is Harrell’s C-Index [24], which is a goodness-of-fit measure for models producing risk scores widely used in survival analysis. Mathematical formulations of the penalized Cox model, cross-validation procedure, and Harrell’s C-index are provided in Appendix 1.

### Function *cv.glmnet*

The simulations utilize the R package *glmnet*, which allows the specification of generalized linear models regularized with Lasso, Ridge, and elastic net penalties, including Cox models as a part of the generalized linear model family. The optimization process employs cyclical coordinate descent in a pathwise fashion [25]. Of relevance is the *cv.glmnet* function, which determines an optimal parameter λ in a penalized Cox model through CV [25, 26]. The function enables the specification of hyperparameters, with a primary focus on two crucial parameters: *alpha* ($\:{\upalpha\:}$), the elastic net tuning parameter belonging to the interval [0,1], where the Lasso penalty corresponds to a value of 1, and the Ridge penalty corresponds to 0 (Appendix 1); *nfold*, the number of folds for k-fold CV, with the default value of *k* set to 10. While λ controls the overall strength of shrinkage applied to the regression coefficients, α determines the relative contribution of Lasso and Ridge penalties, influencing sparsity versus coefficient stabilization. The optimal Lasso coefficients are determined on the basis of λ, where the CV curve reaches its minimum.

### Folds in CV

An important parameter that can potentially impact the outcomes of Lasso-CV is the number of folds *k*, which represents the number of groups into which a data sample is divided during the resampling process. An inappropriate choice of the number of folds may lead to either high variance or high bias [27]. While the default value for *k* in the *cv.glmnet* is 10, we opted to explore the effects of varying this parameter on the results.

To assess the distinctiveness of Lasso-CV models with different fold configurations, we conducted simulations using 100 random seeds for a range of folds {3, 5, 10, 15, 20}. This investigation aims to provide insights into the impact of altering the number of folds on the behavior and performance of the Lasso-CV models.

### Stratification

We investigated the potential efficacy of stratification, as a means of extending the Cox model to accommodate nonproportionality concerning certain covariates [28], to reduce the variability observed in the Lasso-CV results. Stratification allows the baseline hazard to vary across levels of the violating covariate, thereby avoiding estimation of a single overall proportional hazard effect. Predictors satisfying the proportional hazards assumption were included as covariates, whereas those violating the assumption were used as stratifying variables and excluded from the penalized predictor set. Potential stratification variables were identified by assessing proportional hazards violations using scaled Schönfeld [29], via the *cox.zph* function in R [30, 31] with a significance threshold of 0.05.

During the simulation process, we compared stability metrics (model size, λ value, and C-index) between the full model, where all predictors were included without stratification, and a series of candidate models incorporating different stratification strategies. These included models in which one violating covariate at a time was replaced by its stratified version, as well as models applying combined stratification of variables violating the proportional hazards assumption (dual, triple, and fully stratified scenarios). In all cases, stratifying variables defined separate baseline hazards but were not penalized. Owing to computational complexity, 50 simulations were performed for each setting to assess the impact of stratification on model stability. Computation times for the candidate stratified models are summarized in Appendix 2.

### Elastic net

Recognizing the increasing prominence of variable selection in contemporary data analysis, Zou and Hastie advocated for Lasso and its sparsity as an effective tool. However, they identified certain limitations, such as the algorithm’s inclination to arbitrarily select only one variable from groups with high pairwise correlations. Additionally, they noted that when the number of observations exceeded the number of predictors (*n* > *p*), Ridge regression outperformed Lasso [2].

In response to these shortcomings, they proposed the (naïve) elastic net as a compromise between Ridge and Lasso, aiming to address the challenges posed by correlated variables and the superiority of Ridge regression in certain scenarios. The elastic net, positioned as a method surpassing Lasso while maintaining comparable sparsity, employs a regularization technique that combines L1 (sum of absolute values of coefficients) and L2 (sum of squared coefficients) penalties. The mixing parameter α determines the balance between the Ridge and Lasso shrinkage approaches and can be specified in the *cv.glmnet* function. We conducted 100 simulations each for α-values ranging from 0 (Ridge) to 1 (Lasso) with increments of 0.1. The evaluation of model stability required different metrics due to the involvement of Ridge regression, which does not perform variable selection. The model size and predictive performance were visually inspected, and the standard deviation (SD) of the model size was replaced with the sum of coefficient variances (SCV) to ensure comparability across all settings.

## Results

### Motivating example

For the mortality data, Lasso-CV not only included many more variables than BBE did on average but also, depending on different random seeds (500 simulations), the number of variables left in the final model differed between 13 and 20 (Table 1). Therefore, in this case, Lasso-CV often hardly did any selection. The discrepancies became even stronger for the CE data, where the number of variables retained in the Lasso-CV model fluctuated between 3 and 16, indicating a complete lack of consistency in variable selection.

**Table 1:** Comparison of Coefficient Estimates and Variable Selection between BBE and Lasso-CV in Cox Proportional Hazards Models for mortality and a composite endpoint

Nr	Variable	Coefficient					
		Mortality (*N* = 882)			Composite endpoint (*N* = 638)		
		BBE	Lasso-CV		BBE	Lasso-CV	
			rs: 18	rs: 185		rs: 1	rs: 3
1	Age, per 5 years	**0.499**	**0.423**	**0.488**	**0.363**	**0.226**	**0.356**
2	Anemia	(-)	**0.269**	**0.416**	(-)	(-)	**0.125**
3	Antihypertensive medication	(-)	(-)	**-0.046**	(-)	(-)	**0.006**
4	BMI, kg/m^2^	**-0.097**	**-0.055**	**-0.095**	**-0.064**	(-)	**-0.057**
5	Charlson comorbidity index	**0.101**	**0.074**	**0.098**	**0.095**	**0.050**	**0.093**
6	Cholesterol level, mg/dL	(-)	(-)	**-0.002**	(-)	(-)	(-)
7	Serum creatinine, per 0.3 mg/l	(-)	(-)	**-0.051**	(-)	(-)	**-0.125**
8	Cystatin C, per 0.3 mg/dl	**0.369**	**0.189**	**0.337**	(-)	**0.073**	**0.207**
9	Diastolic blood pressure, mmHg	(-)	**-0.001**	**-0.002**	(-)	(-)	**0.010**
10	eGFRBIS2, ml/min/1.73m^2^	(-)	(-)	**0.013**	(-)	(-)	(-)
11	HDL cholesterol, mg/dL	(-)	(-)	**-0.005**	(-)	(-)	(-)
12	Hemoglobin, g/dL	(-)	(-)	**0.010**	(-)	(-)	(-)
13	LDL cholesterol mg/dL	(-)	(-)	**0.005**	(-)	(-)	**0.001**
14	log(ACR)	(-)	**0.131**	**0.183**	(-)	(-)	**0.125**
15	log(AZGP1)	**-0.766**	**-0.176**	**-0.737**	**-0.771**	(-)	**-0.722**
16	log(CRP)	(-)	**0.171**	**0.278**	(-)	(-)	**0.302**
17	Male sex	(-)	**0.082**	**0.028**	(-)	(-)	**-0.160**
18	Smoking ever	**0.395**	**0.193**	**0.382**	(-)	(-)	**0.159**
19	Systolic blood pressure, mmHg	(-)	**-0.002**	**-0.005**	(-)	(-)	**-0.006**
20	Waist to hip ratio, per 0.1 units	(-)	**0.012**	**0.174**	(-)	(-)	**0.297**
Number of selected variables		6	13	20	5	3	16
Concordance index (SE)		0.766 (0.016)	0.767 (0.018)	0.769 (0.020)	0.693 (0.020)	0.685 (0.018)	0.694 (0.009)

Five hundred bootstrap samples were used for BBE; 10 folds for LASSO-CV

Columns labeled “rs:” represent illustrative results obtained using arbitrarily selected random seeds and are provided to demonstrate variability in model selection. These examples are not part of the repeated simulation experiments described later in the manuscript

Variables not selected by the model are marked with “(-)”

*BBE* Bootstraped Backward Elimination, *rs *random seed, *Lasso-CV *cross-validated Lasso, *BMI *body mass index, *ACR *urine albumin to creatinine ratio, *AZGP1 *zinc-alpha-2-glycoprotein, *CRP *C-reactive protein

**Fig. 1:**
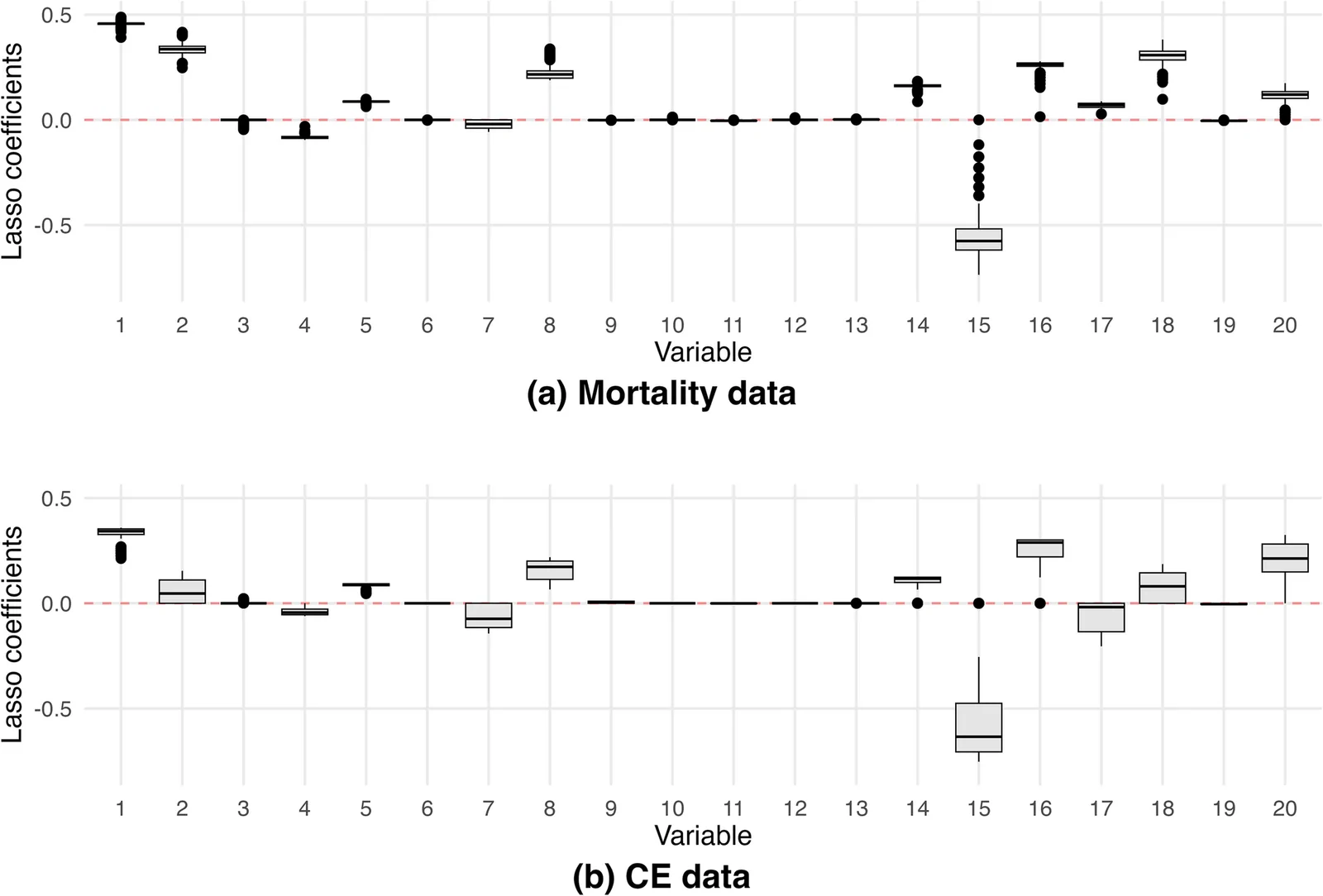
Box plots of the Lasso-CV coefficients of the 20 variables considered for (**a**) mortality and a (**b**) composite endpoint (10 folds for both datasets as a default, random seeds: 1-500) Note: Numbers on the plot correspond to variables listed and numbered identically in Table 1. The red dashed line indicates the zero-reference level (variable exclusion)

We observed considerable variability in the estimated coefficients across simulations (Fig. 1). The box plots reveal generally similar patterns of variable selection across both datasets, with some coefficients consistently shrunk to zero. However, the CE model shows wider boxes, reflecting greater variability and less stable coefficient estimates. The 15th variable (logAZGP1) stands out by occasionally being shrunk to zero, depending on the random seed, even though it has the largest absolute median coefficient overall.

### Folds in CV

For optimal stability in Lasso-CV outcomes, the 10-fold configuration was the most effective for mortality data (Table 2). This is evident in the minimal SD of the model size, coupled with the retention of the optimal λ and C-index. Increasing the number of folds from 3 to 10 reduces the variability in model size. However, further increasing the folds to 15 and 20 results in a rise in variability again, suggesting that the 10-fold setting provides the most stable results in this context.

**Table 2:** Characteristics of the Lasso-CV model across different numbers of folds in the CV (100 simulations each)

	Mortality			Composite endpoint		
	Model size	Lambda	C-index	Model size	Lambda	C-index
3 folds	15.17 (1.93)	0.011 (0.008)	0.765 (0.005)	10.45 (5.27)	0.026 (0.026)	0.686 (0.007)
5 folds	15.52 (1.31)	0.009 (0.005)	0.767 (0.004)	11.49 (4.53)	0.019 (0.020)	0.689 (0.005)
10 folds	**15.88 (0.97)**	0.007 (0.003)	0.769 (0.004)	11.87 (4.50)	0.017 (0.019)	0.691 (0.006)
15 folds	15.97 (1.47)	0.007 (0.005)	0.770 (0.005)	**12.95 (3.67)**	0.011 (0.013)	0.693 (0.007)
20 folds	16.16 (1.53)	0.007 (0.004)	0.770 (0.005)	13.00 (3.72)	0.012 (0.013)	0.694 (0.007)

The results are presented as the means (SDs). Model sizes with the lowest SDs are marked in bold for each outcome dataset

In the case of the CE data, the optimal choice is less apparent. Both the 15-fold and 20-fold scenarios exhibit a modest SD of the model size and high-performance scores (Table 2). In instances of such a “tie”, the decision may be influenced by possibly favoring the smallest model size and SD, which advocates for the 15-fold setting.

As the number of folds increases, the C-index also tends to rise. This pattern is consistent with the expectation that using more folds allows the model to be trained on a larger portion of the data in each iteration, leading to more stable evaluation and reduced sensitivity to specific data splits.

### Stratification

We used Schönfeld residuals to test the proportional hazards assumption and identify covariates with nonproportional effects. Four variables in the mortality data (anemia, *p* = 0.011; Charlson comorbidity index (CCI), *p* = 0.038; hemoglobin, *p* = 0.010; C-reactive protein as log(CRP), *p* = 0.011) and one variable in the CE data (log(CRP), *p* = 0.035) did not satisfy the proportional hazards assumption at the 0.05 significance level. Concerned continuous variables were subsequently categorized using tertile or quartile splits, and the finally selected split was based on the lowest model size variability (see Appendix 3). Specifically, the CCI score was categorized using quartiles, whereas hemoglobin and log(CRP) were categorized using tertiles. This approach reduced the number of baseline hazard strata and supported more stable model estimation. These findings also highlight that commonly used discretization approaches, such as quartile splits, should not be applied automatically, as alternative categorizations (e.g., tertiles) may provide more stable model behaviour depending on the data structure.

The application of stratification in the mortality analysis showed that only for anemia and CCI model stability improved, whereas for hemoglobin and log(CRP) variability remained similar. Model efficiency was otherwise comparable across approaches, although stratification by the CCI resulted in the largest decrease in C-index. In the CE data, stratification by log(CRP) reduced model size but did not reduce its variability (Table 3). For the Lasso-CV model with combined stratification of variables violating the proportional hazards assumption (dual, triple, and fully stratified scenarios), combinations of CCI × hemoglobin and of hemoglobin × log(CRP) were associated with improved model stability (Appendix 4). For some stratification combinations, the model did not converge due to extremely small event counts within one or more strata.

**Table 3:** Characteristics of the Lasso-CV model depending on the stratifying variable that does not satisfy the proportional hazard assumption (50 simulations each, 10-fold CV for mortality and 15-fold CV for CE)

	Mortality (k = 10)			Composite endpoint (k = 15)		
	Model size	Lambda	C-index	Model size	Lambda	C-index
Full model	15.88 (0.98)	0.008 (0.004)	0.768 (0.004)	12.20 (4.03)	0.014 (0.015)	0.693 (0.007)
Anemia	**14.40 (0.57)**	0.009 (0.003)	0.767 (0.004)	-	-	-
CCI	**14.40 (0.73)**	0.009 (0.004)	0.756 (0.004)	**-**	-	-
Hemoglobin	16.08 (1.01)	0.006 (0.003)	0.768 (0.004)	-	-	-
log(CRP)	14.86 (0.93)	0.007 (0.003)	0.767 (0.004)	11.06 (4.15)	0.016 (0.015)	0.691 (0.007)

The results are presented as the means (SDs). Model sizes with meaningfully lower SDs than the full model are marked in bold for each outcome dataset. The choice of the number of folds was based on which number led to optimal stability in Lasso-CV outcomes (see Sect. "Folds in CV"). Stratifying variables were derived from categorical predictors where applicable (e.g., anemia). Continuous or highly granular variables were categorized prior to stratification using tertile or quantile splits, selected based on lower model size variability (see Appendix 3). Specifically, the CCI score was categorized using quantiles, whereas hemoglobin and log(CRP) were categorized using tertiles

*k* number of folds, *CCI *Charlson Comorbidity Index, *CRP *C-reactive protein

### Elastic net

**Fig. 2:**
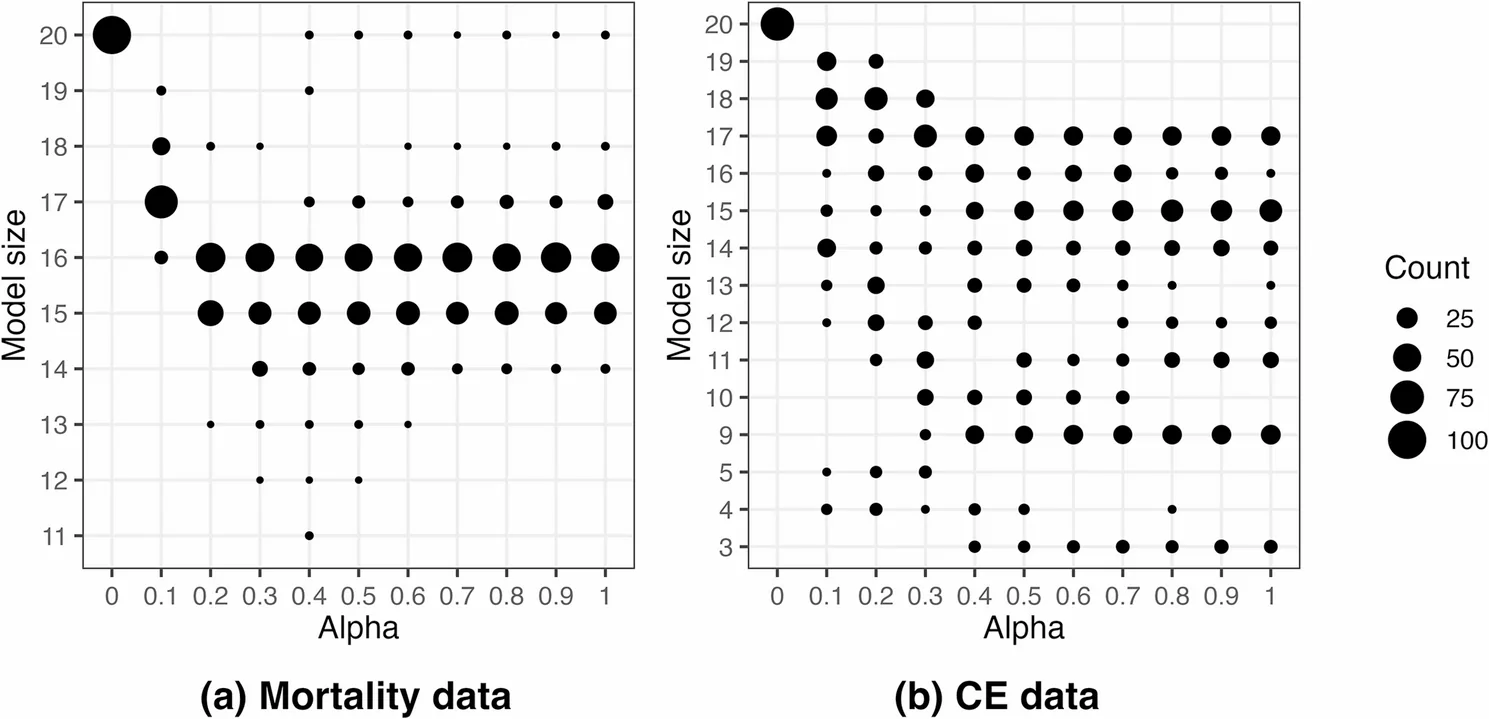
Model sizes selected by elastic net depending on the alpha parameter setting for (**a**) mortality and a (**b**) composite endpoint (100 simulations for each α increment; 10-fold CV for mortality data and 15-fold CV for CE data) Note: The choice of the number of folds was based on which number led to optimal stability in Lasso-CV outcomes (see Sect. "Folds in CV")

As the α parameter increases from 0 (Ridge) toward 1 (Lasso), the models generally become sparser (Fig. 2). However, for α values above 0.5, the reduction in model size becomes less pronounced. In particular, α = 0.4 appears to be the most unstable setting, exhibiting the highest range of model sizes. While the most frequently selected model size for the mortality data contains 16 predictors, for the CE data, it fluctuates between 9, 15, and 17.

**Fig. 3:**
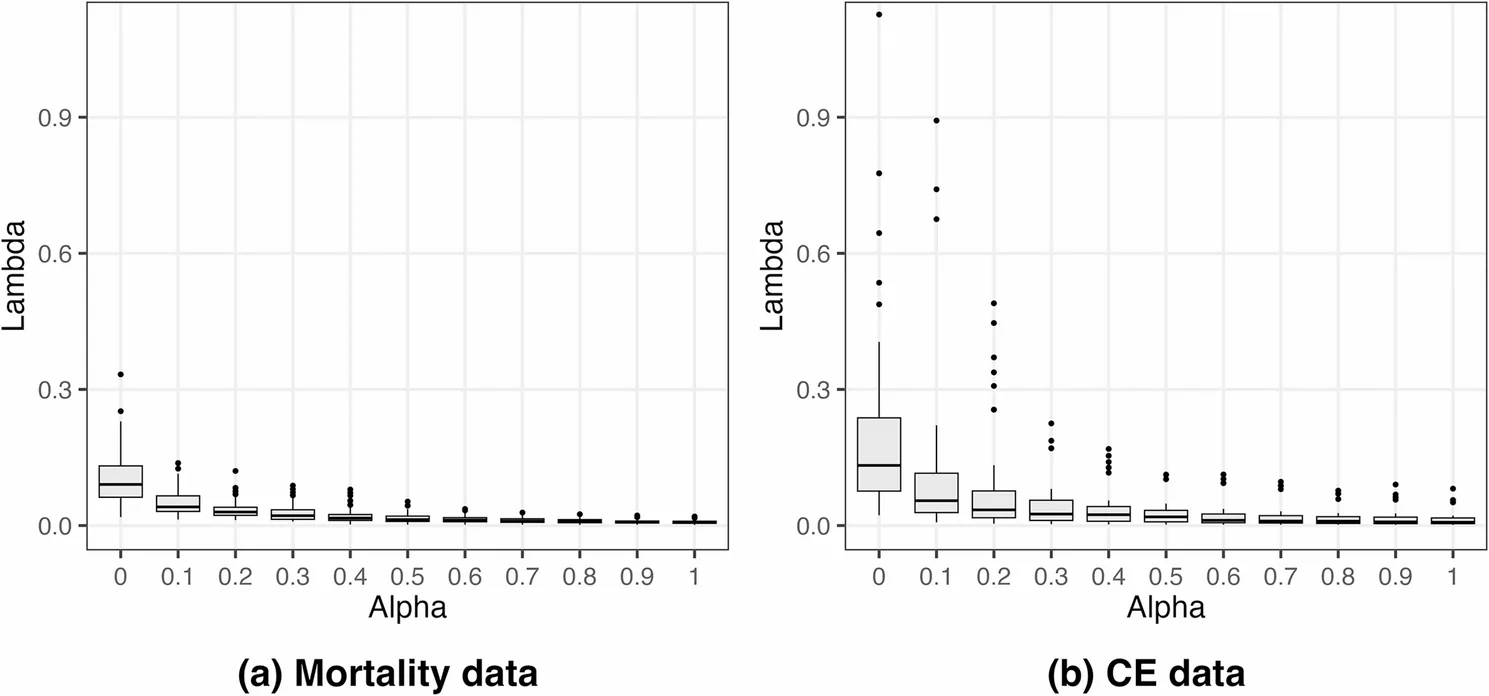
Penalty parameters λ used by the elastic net depending on the alpha parameter setting for (**a**) mortality and a (**b**) composite endpoint (100 simulations for each α increment; 10-fold CV for mortality data and 15-fold CV for CE data) Note: Changes in α alter the balance between sparsity and stability, while λ determines the degree of shrinkage within each configuration. The choice of the number of folds was based on which number led to optimal stability in Lasso-CV outcomes (see Sect. "Folds in CV")

Figure 3 shows that as α increases, both the values and range of λ decrease exponentially. Ridge regression yields more variable shrinkage than Lasso does, with CE coefficients nearly three times larger than those for mortality, reflecting greater instability.

**Fig. 4:**
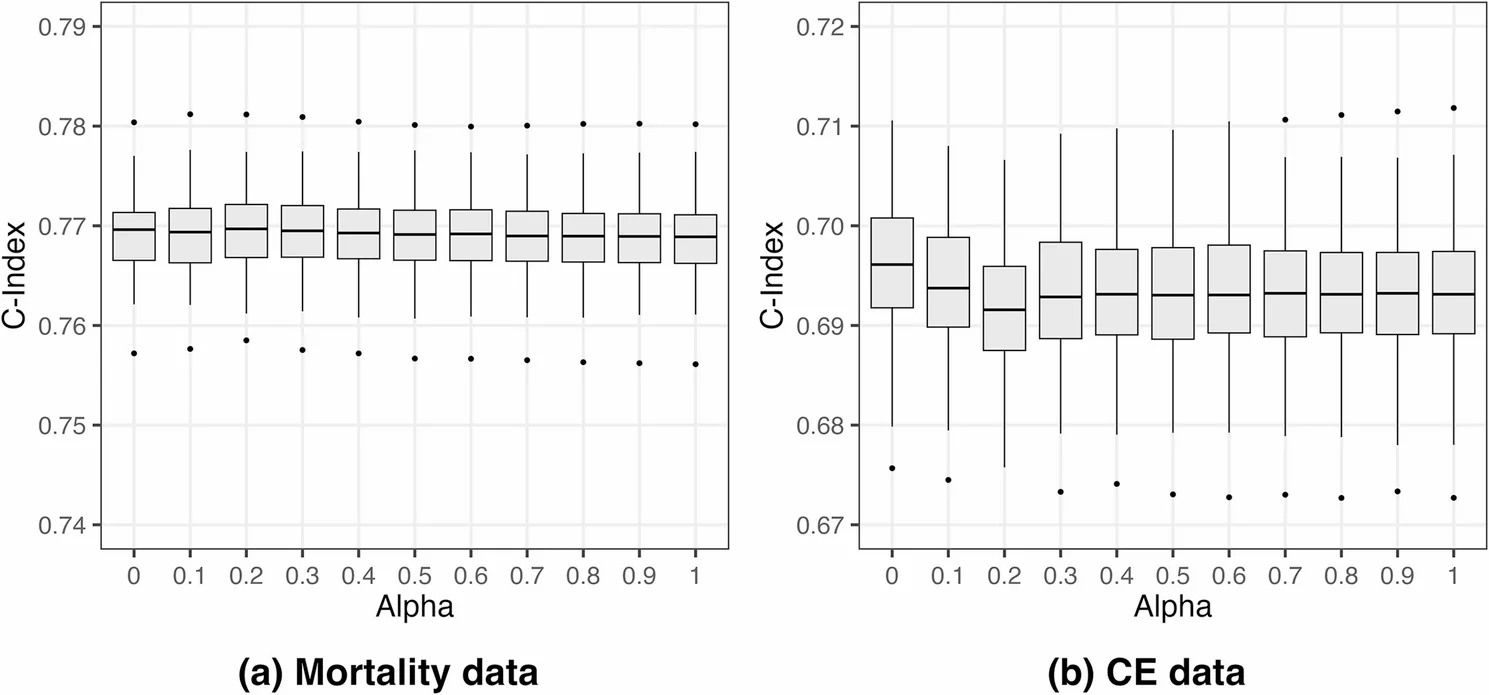
Predictive performance achieved by the elastic net depending on the alpha parameter setting for (**a**) mortality and a (**b**) composite endpoint (100 simulations for each α increment; 10-fold CV for mortality data and 15-fold CV for CE data) Note: The y-axes show different value ranges. The choice of the number of folds was based on which number led to optimal stability in Lasso-CV outcomes (see Sect. "Folds in CV")

**Fig. 5:**
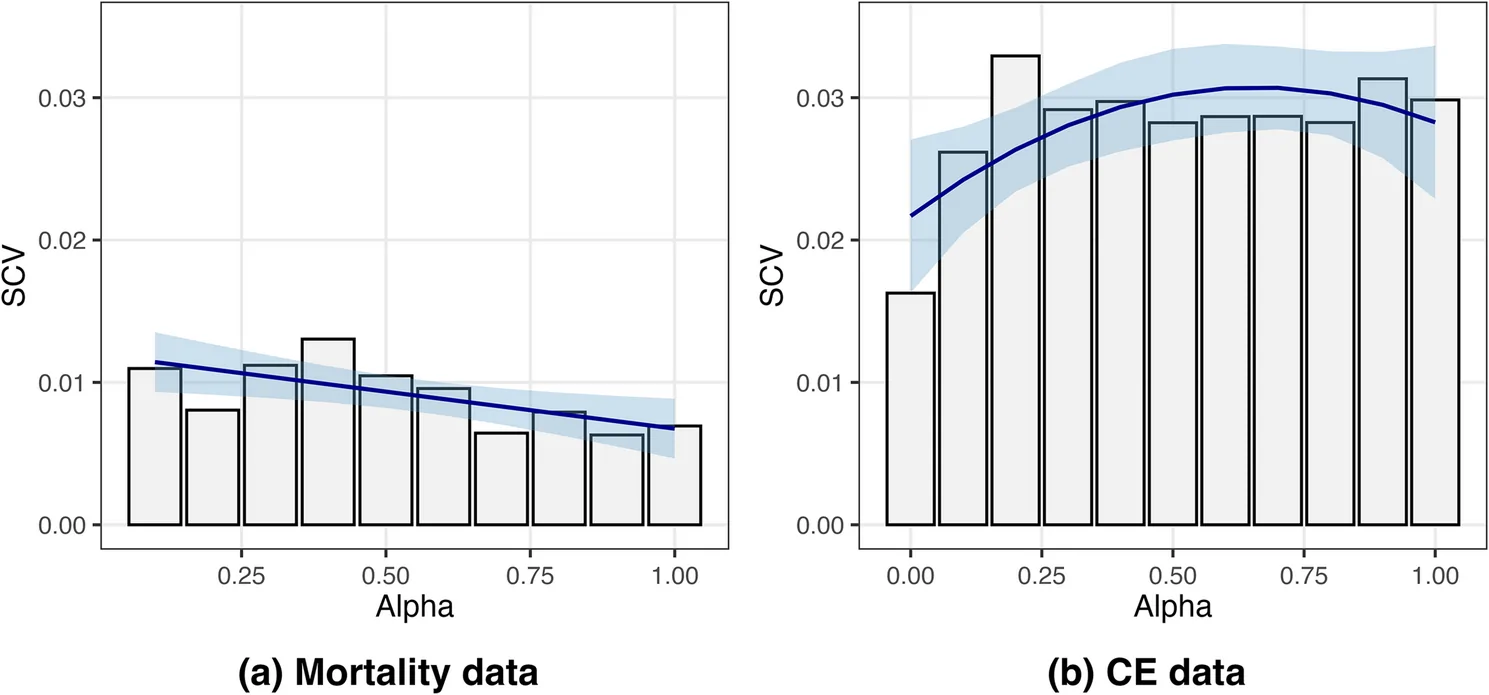
The sum of coefficient variances (SCV) produced by the elastic net depending on the alpha parameter setting for (**a**) mortality and a (**b**) composite endpoint (100 simulations for each α increment; 10-fold CV for mortality data and 15-fold CV for CE data) Note: The dark blue line shows the polynomial fit from a generalized linear model, and the shaded area indicates the 95% confidence interval. The choice of the number of folds was based on which number led to optimal stability in Lasso-CV outcomes (see Sect. "Folds in CV")

The predictive performance (C-index) was similar across the α values, with Ridge being slightly better for the CE (Fig. 4). Model stability (SCV) decreased with α for mortality but plateaued for CE after an initial rise (Fig. 5). Overall, the elastic net with α ≥ 0.6 provided stable and sparse solutions, whereas α = 0.2 occasionally improved mortality performance but performed worst in the CE. Lasso remained a reliable solution in both datasets.

## Discussion

The inconsistency in cross-validated penalized Cox models, observed during predictor selection in our mortality and CE data, was not only dependent on the algorithm’s random seed: our simulation study has shown that the choice of number of folds in the CV procedure, as well as stratifying by a single or multiple covariates not satisfying the proportional hazards assumption, can contribute to improving Lasso’s consistency.

An essential hyperparameter influencing the consistency of Lasso was the number of folds in the CV procedure for determining the optimal value of λ. The simulations revealed that this choice substantially impacts the model’s size, its consistency (measured by the SD of the model’s size), and predictive performance (represented by the C-index). The optimal number of folds, enhancing result consistency, was found to be 10 for mortality data and 15 for CE data. Thus, increasing the number of folds seemed beneficial for more coherent results in unstable data selection settings. However, the substantial dependence of the results on random assignment could also be seen as a drawback of CV in the selection of optimal parameters in the Lasso procedure.

Extending Cox models and stratifying by a single or multiple covariates that did not satisfy the proportional hazards assumption reduced variability in Lasso results in selected scenarios, sometimes with a modest loss in predictive performance. In the mortality data, two out of four stratifying variables improved stability over the full model, and two combined stratification strategies. In the CE data, stratifying the only covariate violating the assumption did not enhance model consistency. Thereby, considering different quantile splits for continuous variables could also be of benefit. A limitation is the high computational time required to run multiple simulations of stratified models.

When using the elastic net, a regularization method that combines L1 and L2 penalties, it seemed advisable to avoid using a mixing parameter smaller than 0.6 on the basis of our data. However, across various scenarios, the elastic net did not substantially surpass Lasso in terms of the variability of the model’s size and predictive performance for both endpoints. Therefore, evaluating its performance on a specific dataset is advisable. Given the distinctive features that set Ridge and Lasso methods apart and the inherent trade-off between sparsity and stability in learning algorithms, explicit conclusions favoring one over the other cannot be drawn. While a comparison between Lasso and elastic net is more straightforward, as both serve as variable selection methods, the choice remains ambiguous and depends on specific criteria to determine the advantage of one over the other.

When comparing our research with existing studies, most investigations into the instability of penalized Cox models have focused on high-dimensional genomic settings, which differ from our clinical, low-dimensional context. One relevant study of low-dimensional data examined the effect of the shrinkage parameter on model stability, but in the context of linear regression [32]. This highlights broader concerns about regularization methods.

Moreover, numerous new methodologies, derived as modified versions of Lasso, have emerged in the last decade. It would be beneficial to systematically compare these methods and assess their capacity to produce robust results that are less prone to challenges related to cross-validation and the careful selection of tuning parameters and provide guidance to users of these methods.

### Strengths and limitations

This study evaluated numerous scenarios of adjusting the setting of the Lasso CV and its implications for the consistency of variable selection. Particularly when simulating numerous Lasso models, increasing the number of folds in the CV or implementing stratification notably increased the time required for simulations. Because the investigation focuses on resampling-based variability rather than synthetic data generation, the findings should be interpreted as reflecting algorithmic behavior under these specific clinical modelling conditions.

Evaluating the predictive performance (C-index) on the same observations used to train a model could have led to overfitting. Furthermore, we did not partition the data into training and test sets, as investigating predictive performance was of secondary importance and primarily served informative purposes, since Cox models are not commonly employed for actual predictions [26]. The C-index served as a benchmark when explicit determination or ambiguity in the comparison of stability results was challenging. Secondly, random assignment into training and test sets would have introduced an additional layer of randomness, potentially further destabilizing Lasso results and complicating the differentiation from the randomness associated with fold assignment in CV.

In contrast to other studies mentioned in the introduction that investigated sensitivity to random fold assignment in simulated data, this paper focuses on a specific situation where the compared datasets primarily differ concerning the response variable (mortality or CE) and the number of cases. From this perspective, the greater instability of the results for CE than for mortality may also stem from the smaller sample size.

### Summary

To our knowledge, our study is the first to systematically investigate instability in Lasso-penalized Cox models applied to low-dimensional clinical data. This makes our work novel and also raises the need to explore how increasing the number of predictors affects model stability across both low- and high-dimensional settings.

This study shows that the consistency of Lasso in penalized Cox models is sensitive to the number of cross-validation folds and can be improved by increasing this number, although at the cost of computational time. Stratification by covariates violating the proportional hazards assumption can further enhance stability, yet it is also time-intensive. Elastic net offered no clear advantage over Lasso in our settings. Overall, these findings underscore the need for careful tuning and highlight opportunities for developing more stable and efficient methods in time-to-event modeling.

## Supplementary Information

Supplementary Material 1.

## Data Availability

The datasets analyzed during the current study are not publicly available due to restrictions in the patient consent form, but are available from the corresponding author on reasonable request.
