# Supplementary material for: Investigating the instability of variable selection in Lasso-penalized Cox proportional hazards models: a simulation study on low-dimensional real-world medical data
Source: BMC Med Res Methodol. 2026 Oct 24;26:241. doi: 10.1186/s12874-026-03019-4 (PMC13749795; doi:10.1186/s12874-026-03019-4)
Supplement: Supplementary file 1 [file 12874_2026_3019_MOESM1_ESM.docx]

**Supplementary Information**

**Investigating the Instability of Variable Selection in Lasso-Penalized Cox Proportional Hazards Models: A Simulation Study on Low-Dimensional Real-World Medical Data**

**Appendix 1**. Mathematical formulation of penalized Cox models and cross-validation

**Appendix 2.** Computation time for candidate stratified models in seconds (50 simulations each; minutes in parentheses)

**Appendix 3. Characteristics of the Lasso-CV model across stratifications for variables violating the proportional hazards assumption, in original for binary or tertile- and quartile-based splits for continuous variables (50 simulations each, 10-fold CV for mortality and 15-fold CV for CE).**

**Appendix 4. Characteristics of the Lasso-CV model under combined stratification of variables violating the proportional hazards assumption (dual, triple, and full stratification scenarios; 50 simulations each).**

**Appendix 1**. Mathematical formulation of penalized Cox models and cross-validation

***Penalized Cox proportional models***

The Cox proportional hazards model assumes

$$h\left( t|X \right)= h_{0}\left( t \right)\exp\left( X\beta\right),$$

where $X$denotes the vector of predictors and $\beta$ the corresponding regression coefficients; $h_{0}\left( t \right)$ is the baseline hazard function.

Penalized Cox regression estimates coefficients by maximizing the penalized partial likelihood

$$\hat{\beta}=\arg\max_{\beta} \left\{ \mathcal{l}\left( \beta\right)-P_{\lambda}(\beta) \right\},$$

where $\mathcal{l}\left( \beta\right)$ is the Cox partial log-likelihood and $P_{\lambda}\left( \beta\right)$ is a penalty term.

For Lasso regression,

$$P_{\lambda}\left( \beta\right)=\lambda\sum_{j=1}^{p} \left| \beta_{j} \right|,$$

where $p$ is the number of predictors, $\lambda\geq0$ is the tuning parameter controlling the strength of penalization, and $\left| \beta_{j} \right|$ represents the absolute value of $j$*-*th coefficient.

For elastic net regression,

$$P_{\lambda}\left( \beta\right)=\lambda\left( \alpha\sum_{j=1}^{p} \left| \beta_{j} \right|+\frac{1-\alpha}{2}\sum_{j=1}^{p} \beta_{j}^{2} \right),$$

where $\alpha\in\left[ 0, 1 \right]$is the mixing parameter controlling the balance between Lasso and Ridge penalties. When $\alpha=1$, the model corresponds to Lasso regression, and when $\alpha=0$, it reduces to Ridge regression. The term $\sum_{j=1}^{p} \left| \beta_{j} \right|$ corresponds to the $L_{1}$penalty (Lasso) and promotes sparsity by shrinking some coefficients to zero, whereas $\sum_{j=1}^{p} \beta_{j}^{2}$ represents the squared $L_{2}$ penalty (Ridge), which stabilizes coefficient estimates, particularly in the presence of correlated predictors.

***Cross-validation and performance measure***

The tuning parameter $\lambda$ was selected using $k$-fold cross-validation. The dataset is partitioned into $k$disjoint folds $D_{1},\ldots,D_{k}$. For each fold $m$, the model is trained on the remaining data $D_{-m}$and evaluated on the hold-out fold $D_{m}$. The cross-validated performance is computed as

$$\mathrm{CV}(\lambda)=\frac{1}{k}\sum_{m=1}^{k} \mathcal{L(}D_{m};\hat{\beta}^{(-m)}(\lambda)),$$

where $\mathcal{L}\left( \cdot\right)$denotes the chosen performance metric and $\hat{\beta}^{(-m)}(\lambda)$ are the coefficients estimated without fold $m$. The optimal tuning parameter is selected as

$$\hat{\lambda}=\arg\max_{\lambda}\mathrm{CV}(\lambda),$$

because Harrell’s C-index is maximized.

***Harrell’s concordance index***

Predictive performance was assessed using Harrell’s C-index, which measures the proportion of concordant risk predictions among comparable subject pairs. It can be written as

$$\hat{C}\left( \hat{\beta} \right)=\frac{\sum_{i,j} I\text{ }\left( \hat{\beta}^{T}X_{i}>\hat{\beta}^{T}X_{j} \right)\text{ }I\left( T_{i}<T_{j} \right)\text{ }\delta_{i}}{\sum_{i,j} I\left( T_{i}<T_{j} \right)\text{ }\delta_{i}},$$

where $X_{i}$ is the vector of predictors for subject $i$; $\hat{\beta}$ are the estimated regression coefficients, $\eta_{i}=\hat{\beta}^{T}X_{i}$ is the linear predictor (risk score), $T_{i}$ is the observed follow-up time, $\delta_{i}\in\{0,1\}$ is the event indicator (1 = event observed, 0 = censored), and $I(\cdot)$ is the indicator function equal to 1 when the condition holds and 0 otherwise. The numerator counts **concordant comparable pairs**, i.e., subject pairs for which the individual with the higher predicted risk experiences the event earlier. The denominator represents the total number of comparable pairs, excluding comparisons where the earlier observation is censored.

***Model specification for mortality and composite endpoint analyses***

For the mortality outcome, the penalized Cox model included the following linear predictor:

$$\eta_{\text{mort}}=\beta_{1}(\text{Age, per 5 years})+\beta_{2}(\text{Male sex})+\beta_{3}(\text{BMI})+\beta_{4}(\text{Waist-to-hip ratio})+\beta_{5}(\text{Smoking ever})+\beta_{6}(\text{Anemia})+\beta_{7}(\text{Systolic blood pressure})+\beta_{8}(\text{Diastolic blood pressure})+\beta_{9}(\text{Serum creatinine})+\beta_{10}(\text{Cystatin C})+\beta_{11}(\text{Hemoglobin})+\beta_{12}(\text{Cholesterol level})+\beta_{13}(\text{HDL cholesterol})+\beta_{14}(\text{LDL cholesterol})+\beta_{15}\log(\text{CRP})+\beta_{16}(\text{eGFRBIS2})+\beta_{17}\log(\text{ACR})+\beta_{18}\log(\text{AZGP1})+\beta_{19}(\text{Charlson comorbidity index})+\beta_{20}(\text{Antihypertensive medication}).$$

An analogous specification was used for the composite endpoint model, with the same set of predictors but using the composite endpoint as the event indicator.

**Appendix 2.** Computation time for candidate stratified models in seconds (50 simulations each; minutes in parentheses)

| **Candidate stratified model** | **Mortality**  (*k*=10) | **Composite endpoint** (*k*=15) |
| --- | --- | --- |
| Full model (without stratification) | 26.01 (0.43) | 28.68 (0.48) |
| Anemia | 295.67 (4.93) | - |
| CCI | 315.83 (5.26) | - |
| Hemoglobin | 442.88 (7.38) | - |
| log(CRP) | 365.52 (6.09) | 319.71 (5.33) |

**Note:** Computation times were obtained on a MacBook Pro equipped with an Apple M1 chip (8-core CPU: 4 performance and 4 efficiency cores) and 16 GB RAM. Hyperparameter *k* indicates the number of folds.

**Appendix 3. Characteristics of the Lasso-CV model across stratifications for variables violating the proportional hazards assumption, in original for binary or tertile- and quartile-based splits for continuous variables (50 simulations each, 10-fold CV for mortality and 15-fold CV for CE).**

|  | **Mortality** (*k* = 10) | | | | | | |
| --- | --- | --- | --- | --- | --- | --- | --- |
|  | **Model size** | **Lambda** | **C-index** | **Model size** | | **Lambda** | **C-index** |
| **Full model** | 15.88 (0.98) | 0.008 (0.004) | 0.768 (0.004) | |  |  |  |
| **Anemia** | 14.40 (0.57) | 0.009 (0.003) | 0.767 (0.004) | |  |  |  |
|  | **Tertile split** | | | | **Quartile split** | | |
| **CCI** | 14.54 (1.03) | 0.009 (0.005) | 0.757 (0.004) | | **14.40 (0.73)** | 0.009 (0.004) | 0.756 (0.004) |
| **Hemoglobin** | **16.08 (1.01)** | 0.006 (0.003) | 0.768 (0.004) | | 16.08 (1.05) | 0.006 (0.004) | 0.767 (0.004) |
| **log(CRP)** | **14.86 (0.93)** | 0.007 (0.003) | 0.767 (0.004) | | 14.96 (1.09) | 0.007 (0.003) | 0.766 (0.004) |
|  | **Composite endpoint** (*k* = 15) | | | | | | |
|  | **Model size** | **Lambda** | **C-index** | **Model size** | | **Lambda** | **C-index** |
| **Full model** | 12.20 (4.03) | 0.014 (0.015) | 0.693 (0.007) |  | |  |  |
|  | **Tertile split** | | | **Quartile split** | | | |
| **log(CRP)** | **11.06 (4.15)** | 0.016 (0.015) | 0.691 (0.007) | 10.44 (4.47) | | 0.018 (0.016) | 0.690 (0.007) |

Note: k, number of folds; CCI, Charlson Comorbidity Index; CRP, C-reactive protein. The results are presented as the means (SDs). The full model without stratification is shown for comparison, and anemia is a binary variable that was used in the original for stratification. Bolded values indicate the preferred split based on model size variability.

**Appendix 4. Characteristics of the Lasso-CV model under combined stratification of variables violating the proportional hazards assumption (dual, triple, and full stratification scenarios; 50 simulations each).**

|  | **Mortality** (*k* = 10) | | |  |
| --- | --- | --- | --- | --- |
|  | **Model size** | **Lambda** | **C-index** | **Min. n**  **(mortality = 1)** |
| Full model | 15.88 (0.98) | 0.008 (0.004) | 0.768 (0.004) | NA |
| Anemia × CCI | 12.58 (1.50) | 0.010 (0.003) | 0.752 (0.004) | 4 |
| Anemia × log(CRP) | 13.08 (1.31) | 0.009 (0.004) | 0.764 (0.004) | 12 |
| Anemia × hemoglobin | 14.04 (1.01) | 0.008 (0.004) | 0.767 (0.004) | 33 |
| CCI × hemoglobin | **13.72 (0.57)** | 0.007 (0.003) | 0.757 (0.004) | 3 |
| CCI × log(CRP) | 12.66 (1.17) | 0.009 (0.004) | 0.754 (0.005) | 3 |
| Hemoglobin × log(CRP) | **14.26 (0.72)** | 0.005 (0.002) | 0.767 (0.004) | 16 |
| Anemia × hemoglobin × log(CRP) | 13.10 (1.33) | 0.007 (0.004) | 0.765 (0.004) | 9 |
| Anemia × CCI × log(CRP) | - | - | - | 0 |
| Anemia × CCI × hemoglobin | **-** | - | - | 2 |
| CCI × hemoglobin × log(CRP) | **-** | - | - | 0 |
| Anemia × hemoglobin × log(CRP) × CCI | **-** | - | - | 0 |

Note: k, number of folds; CCI, Charlson Comorbidity Index; CRP, C-reactive protein. The results are presented as the means (SDs). “Min. n” represents the minimum number of mortality events observed within any stratum for a given stratification combination. Models marked with “-“ did not converge due to extremely small event counts within one or more strata. Bolded values indicate lower variability in model size compared to the full model. The CCI score was categorized using quartiles, hemoglobin and log(CRP) were categorized using tertiles.
